# MEF2C and HDAC5 regulate *Egr1* and *Arc* genes to increase dendritic spine density and complexity in early enriched environment

**DOI:** 10.1042/NS20190147

**Published:** 2020-07-23

**Authors:** Shu Juan Puang, Bavani Elanggovan, Tendy Ching, Judy C.G. Sng

**Affiliations:** 1 Department of Pharmacology, Yong Loo Lin School of Medicine, National University of Singapore, Singapore; 2 Lee Kong Chian School of Medicine, Nanyang Technological University, Singapore; 3 Integrative Neuroscience Programme, Singapore Institute for Clinical Sciences, Agency for Science and Technology (A*STAR), Singapore

**Keywords:** environmental enrichment, epigenetics, experience dependent plasticity, HDAC5, MEF2C, synaptic plasticity genes

## Abstract

We investigated the effects of environmental enrichment during critical period of early postnatal life and how it interplays with the epigenome to affect experience-dependent visual cortical plasticity. Mice raised in an EE from birth to during CP have increased spine density and dendritic complexity in the visual cortex. EE upregulates synaptic plasticity genes, *Arc* and *Egr1*, and a transcription factor MEF2C. We also observed an increase in MEF2C binding to the promoters of *Arc* and *Egr1*. In addition, pups raised in EE show a reduction in HDAC5 and its binding to promoters of *Mef2c, Arc* and *Egr1* genes. With an overexpression of *Mef2c*, neurite outgrowth increased in complexity. Our results suggest a possible underlying molecular mechanism of EE, acting through MEF2C and HDAC5, which drive *Arc* and *Egr1.* This could lead to the observed increased dendritic spine density and complexity induced by early EE.

## Introduction

Neuronal circuits that process sensory information are shaped by patterns of cellular activity during early brain development called critical periods or CPs [1]. CPs are known to be present in several sensory systems of the brain, such as the barrel representation of whiskers in somatosensory cortex, tonotopic map refinement in auditory cortex and human language acquisition in the Broca’s area but the best-described case is the role of light on the development of the visual system [1–6]

Visual cortical circuits exhibit maximal plasticity during CP, but this plasticity is lost by adulthood [1]. The loss of this extraordinary plasticity may reflect sequential locks placed on a molecular pathway as the visual cortex consolidates into a structurally elaborated circuitry. The critical period in mouse visual cortex has been linked to a specific molecular logic of gene regulation [7–10] and we postulated that epigenetics could help fine-tune gene activity essential for CP plasticity. While the role of epigenetic modifications in brain plasticity has only begun to be understood [11,12], the critical period is an ideal model for the study of experience-dependent epigenetic modifications and their interplay with external environment to affect outcomes later in life.

The visual system is highly amenable and is easily manipulated either by decreasing external sensory experience or by dark rearing and monocular deprivation or increasing external stimuli by environmental enrichment. In the latter paradigm, animals are reared in big cages filled with toys, running wheels, tunnels and nesting materials. Studies have shown that enriched environment (EE) leads to hyperacuity, which enhances their inquisition, social interactions and sensory-motor stimulations [13,14]. Studies have found combination of EE conditions involving motor and visual stimuli can enhance the overall enriched experience [15]. Motor stimulus through running can potentially boost visual plasticity and activity, hence enhancing visual acuity in the visual cortex [16]. At the anatomical level, EE has been shown to augment dendritic branching, spine and synaptic densities and neurogenesis [17,18]. At the circuitry level, studies have demonstrated that EE can initiate maturation of the GABAergic system by the increase of BDNF in the visual cortex, triggering dopamine release and leading to an acceleration of the visual system development [14,19,20]. EE also has a profound effect with vast improvement in motor skills, learning and memory and has been used as rehabilitative therapy in pathological diseases such as Huntington’s disease [21], after stroke [22], Alzheimer’s disease [23] and Down Syndrome [24]. This is further extended to explore EE as a form of preventive measure for cognitive decline and dementia risk [25]. Despite the extensive studies of EE and its enhancement on neuroplasticity, its molecular mechanisms are yet to be unraveled.

In this present study, we ask how environmental stimuli interact with the epigenome to shape cellular function affecting outcomes later in life. We observed that by raising animals exposed to EE from birth to the peak of CP underwent dramatic developmental changes. These animals not only have increased dendritic branching and spine density in the primary visual cortex, but also attained higher visual acuity when tested behaviorally. We sought to unravel the molecular mechanism behind EE that drives these developmental changes. Two candidate synaptic plasticity genes, activity-regulated cytoskeleton-associated protein (*Arc*) and early growth response protein 1 (*Egr1*) and a transcription factor myocyte enhancer-binding factor 2C (*Mef2c*) were up-regulated in animals exposed to EE during CP, and we hypothesized that both *Arc* and *Egr1* genes are regulated by MEF2C. We performed chromatin immunoprecipitation (ChIP) assays and found that MEF2C binds to the promoters of *Arc* and *Egr1* in mice raised in EE during CP. In addition, enriched pups showed a reduction in HDAC5 binding to promoters of *Mef2c, Arc* and *Egr1* genes to allow their expressional increase. Taken together, our results elucidate the underlying molecular mechanism of EE through MEF2C and HDAC5 that drive experience-dependent plasticity in mouse visual cortex.

## Methods

### Animals

All animal protocols have been approved by the Institutional Animal Care and Use Committee (IACUC) at Biopolis Resource Centre, A*STAR, in the Neuroepigenetics laboratory. C56BL/6 mice of mixed sex were maintained on a 12-h light/dark cycle and had access to food and water *ad libitum*. Mixed sex C56BL/6 mice were used in our experiments as we did not notice sex differences. Previous data have also shown no significant sex differences in visual detection, pattern discrimination and visual acuity of C56BL/6 mice strain [26].

### Rearing environments

C57BL6 time-mated dams were placed in SC or EE for 2–4 days prior to the estimated time of delivery and subsequently gave birth in their respective environments. A standard condition (SC) was a basic housing of a 19 × 30 cm shoebox without any form of social stimulation. The enriched environment consists of a 45 × 45 cm cage arena with enhanced living conditions, containing various toys such as running wheels, tunnels, toilet rolls, wood chews, shelters and nesting material*.* The positions of the toys were changed every week and two filler females are part of EE to promote social interaction. The EE condition selected aims to stimulate cognitive, social and sensory-motor developments that provide an overall enriched experience for visual plasticity to take place during critical period [27,28]. The pups were raised in the respective environments with their mothers from birth to the peak of the critical period or postnatal day 28 (PND 0-28). The pups were killed on PND 28 for molecular analyses. Mice were anesthetized by isoflurane, followed by cervical dislocation. Visual cortex tissues were excised under a dissecting microscope and used for protein, RNA extraction and dendritic morphology analysis. Perfused visual cortical tissues were used for ChIP assays.

### Golgi-Cox staining

For each SC or EE condition, three mice from different litters were perfused with 0.1 M phosphate buffer saline (PBS, pH 7.4) followed by 4% paraformaldehyde. The brain was removed and subsequently postfixed in 4% paraformaldehyde for an hour before it was transferred to 30% sucrose in 0.1 M PBS and stored at 4°C overnight in the fixative. The brains were sectioned at 150 μm thickness into 3–4 sections with a cryostat and then stained accordingly to the protocol given in the FD Rapid GolgiStain™ kit (FD Neuro Technologies Inc). Imaging of the dendrites was performed with a confocal laser-scanning microscope (Nikon A1R confocal laser microscope system). Blind to condition, 3D neuronal reconstructions and spine analysis of pyramidal neurons in V1 were quantified by personnel from MBF Labs (MBF Bioscience, Williston, VT, U.S.A.).

### Dendritic reconstruction and analysis

Uniformly impregnated V1 neurons were selected for reconstruction and dendritic analysis. 3D neuronal reconstructions were performed using a modified light microscope (Zeiss AxioImager Z1; Zeiss, Germany) under 100× oil (Plan-Apochromat; 1.4 numerical aperture) controlled by Neurolucida software (v.10.5, MBF Bioscience, Williston VT). The microscope system consisted of an internal Z motor, a motorized specimen stage (Ludl Electronics, Hawthorne, NY, U.S.A.), external focus encoder (Heidenhain, Schaumburg, IL, U.S.A.), and a CCD monochrome video camera (mRm; Zeiss). Neurons were traced in their entirety, matching dendritic diameter and location of dendritic spines. The soma was traced at its widest point in the 2D plane to estimate the cross-sectional area. Neurons that displayed breakages in dendrites were excluded in final analysis. We have also followed the analysis of neurons as described by Faherty, Kerley [29]. Quantitative parameters included dendritic length, spine number and spine density for both the apical dendrite and basolateral dendrites. A total of six neurons (three biological and technical replicates) per treatment were reconstructed.

### Antibodies

Histone deacetylase 5 (HDAC5, Santa Cruz, SC-11419; 2 µg for ChIP; HDAC5, Cell Signaling, 2082; 1:50 for Co-IP); MEF2C (Cell Signaling, 5030; 1:50 for ChIP; 1:1000 for Co-IP WB; 1:500 for ICC WB); TUJ1 (Millipore, MAB1637; 1:1000 for ICC WB); MAP-2 Alexa Fluor 680 (Invitrogen, A21109; 1:3000 for Co-IP WB).

### Real-time quantitative PCR

Total RNA was extracted from 5 SC and 5 EE visual cortex of different litters using RNeasy® Mini kit (Qiagen) and converted to cDNA. Real-time qPCR was done using Taqman primers for *Arc* (Mm00479619_g1), *Egr1* (Mm00656724_m1), *Mef2a* (Mm01318991_m1), *Mef2b* (Mm00484956_g1), *Mef2c* (Mm00600423_m1), *Mef2d* (Mm00504931_m1), *Hdac5* (Mm00515917_m1) and master mix (Applied Biosystems) on the FAST7900HT machine (Applied Biosystems). All analysis were done on the RQ Manager (Applied Biosystems) provided with the machine. Samples were normalized to their respective standard condition. ΔΔCT was calculated with two housekeeping genes: *GAPDH* (Mm99999915_g1) and *β-actin* (Mm02619580_g1). The final fold change is the average of the two values. Technical triplicates were run according to the MICE guidelines [30].

### Co-immunoprecipitation and western blot

Mice were perfused using the same protocol as mentioned in Golgi-Cox staining*.* The frozen tissues were pooled from four pairs of visual cortex from pups of the same dam and homogenized using Pierce IP Lysis buffer (Thermo Scientific) for each experiment respectively (3–4 biological replicates). The lysates were incubated with HDAC5 antibody overnight with rotation at 4°C. The immune-complexes were pulled down with magnetic beads, followed by resuspension in SDS loading buffer. The precipitated proteins were resolved by SDS/PAGE and the immunoblots were blocked with Odyssey blocking buffer (Li-Cor Biosciences) and probed with either anti-HDAC5 or anti-MEF2C followed by detection with Alexa Fluor 680 secondary antibody. The immunoreactive bands were visualized with Odyssey (Li-Cor Biosciences). The intensity of the Western blot protein bands was carried out with the Odyssey application software version 2.1.

### Chromatin immunoprecipitation

The perfused tissues were pooled from 11 pairs of visual cortex (3–4 biological replicates). Each biological replicate constituted to pooled tissue of pups from different litters. They were quenched, lysed and sheared with the Diagenode Bioruptor for 15 cycles of 30-s intervals. Samples were pre-cleared and incubated with MEF2C, HDAC5, and No Antibody, overnight with rotation at 4°C. The immune-complexes were pulled down with magnetic beads, reversed cross-link and purified with phenol–chloroform. Immunoprecipitated chromatin was quantified by real-time quantitative PCR on FAST7900HT (Applied Biosystems) using SYBR-Green master mix (Applied Biosystems), using 10% input. Primers used were: *Egr1* forward, 5′-GTGCCCACCACTCTTGGAT-3′, and reverse, 5′-CGAATCGGCCTCTATTTCAA-3′; *Arc*: forward, 5′-CAGCATAAATAGCCGCTGGT -3′, and reverse, 5′-GAGTGTGGCAGGCTCGTC-3′; *Mef2c*: forward, 5′-TGCAGAAAAGATTCCCACTTG-3′, and reverse, 5′-AGACACTCACAAGGCAAAGAC -3′. Fold enrichment was calculated by adjusting 10% input to 100% (Ct Input – log210) followed by [Dilution Factor (No Antibody)* (100*2∧ (adjusted input -Ct (IP))] to obtain the fold enrichment.

### Cell culture and transduction

P19 cells were differentiated into the neural lineage as described previously. Briefly, cells were differentiated with 1 µM All-Trans Retinoic acid for 4 days and subsequently plated at a density of 2 × 10^5^ cells/well on poly-L-lysine coated glass coverslips in 4- or 12-well plates. One day after plating (Day1), cells were transduced with pre-made lentiviral particles expressing *Mef2c* gene under the suCMV promoter (GenTarget). As a negative control, lentiviral particles with the same lentivector backbone containing a null spacer sequence were used. Seventy-two hours after transduction, protein lysates were collected for Western Blot analysis and dendritic morphology was visualized by immunocytochemistry.

### Primary neuronal culture and transfection

Primary neuronal cultures were prepared from 17- to 18-day-old embryonic mice cerebral cortex. The mice cerebral cortices were dissected immediately after killing in accordance with the guidelines of Institutional Animal Care and Use Committee (IACUC). In brief, the cerebral cortices were incubated with Versene (GIBCO), resuspended in culture media (Neurobasal medium (GIBCO), B27 supplement (GIBCO), 2 mM glutamine, 100 U/ml penicillin and 100 μg/ml streptomycin) and plated at a density 2.5 × 10^5^ cells/cm^2^ on a 0.56 cm^2^ 12-well dish coated with 75 μg/ml poly lysine (ibidi) and/or on a 9.6 cm^2^ 6-well dish (Nunc). To suppress the growth of proliferative cells, 10 μM cytosine arabinoside was added into culture medium from day 2 to 3. Fresh culture media were added every 3 days and maintained at 37°C in a 5% CO_2_/95% O_2_ air-humidified incubator. Subsequently, 25 nM HDAC5 siRNA (Qiagen) was added to the cultures for 24 h. All-Star Negative Control (Qiagen) with a scrambled sequence was used as a negative control. RNA was collected for mRNA expression analysis and dendritic morphology was visualized by immunocytochemistry.

### Immunocytochemistry

Cells grown on coverslips were washed, fixed and blocked for 1 h followed by incubation with anti-MEF2C and anti-TUJ1 antibodies for MEF2C transduction experiments or with anti-HDAC5 and anti-MAP2 for HDAC5 siRNA transfection experiments. They were probed with Alexa-Fluor 488 and 546 secondary antibodies and sealed with ProGold stain. Images were visualized on the confocal laser-scanning microscope (Nikon A1R confocal laser microscope system).

### Statistical analysis

**Figure 1:**
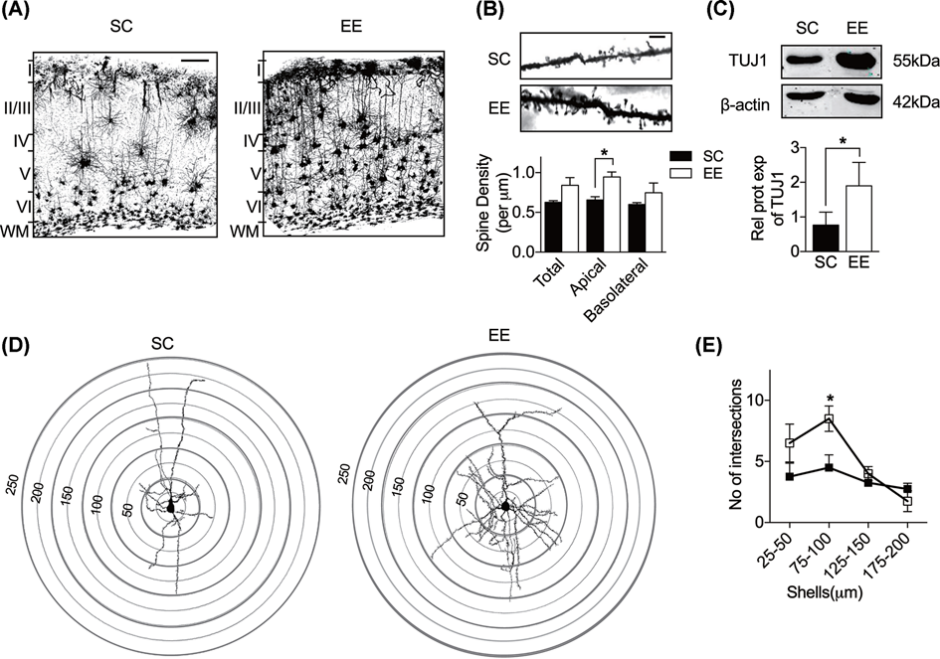
Enriched environment increases dendritic complexity of pyramidal neurons Representative images of Golgi staining in the primary visual cortex of PND28 SC (*n*=3) and PND28 EE (*n*=3) mice, taken at 4× magnification (scale bar=100μm). Enriched environment enhances the dendritic complexity of the pyramidal neurons. (**B**) Photomicrographs of representative Golgi-impregnated pyramidal neurons at the apical dendritic regions of SC and EE taken at 100× magnification (top panel) (scale bar=5μm). Quantification of spine density per μm for the total, apical and basolateral dendrites (bottom panel). Enriched environment increases the total and basolateral spine density with a significant increase in the apical spine density (SC, *n*=3; EE, *n*=3; two-way ANOVA Sidak’s multiple comparisons test). (**C**) Quantification of dendritic marker TUJ1 protein in early EE studied. Immunoblot shows TUJ1 protein enrichment in EE compared to SC. TUJ1 protein expression increases by 2.4-fold (data relative to SC and normalized to β-actin; *n*=4 per group; paired *t* test). (**D**) A representative pyramidal neuron of SC and EE with superimposed concentric circles for Sholl analysis. Concentric circles are drawn at a radius of 25 μm from the center of the soma to 200 μm. (**E**) Quantification of the number of intersections for the respective ranges studied. EE significantly increases the number of intersections for the 75–100 μm range from the soma (SC, *n*=4; EE, *n*=4; two-way ANOVA Sidak’s multiple comparisons test). Black bars represent SC and white bars represent EE. Data are shown as means+S.E.M. and asterisks denote statistical significance; *P<0.05.

**Figure 2:**
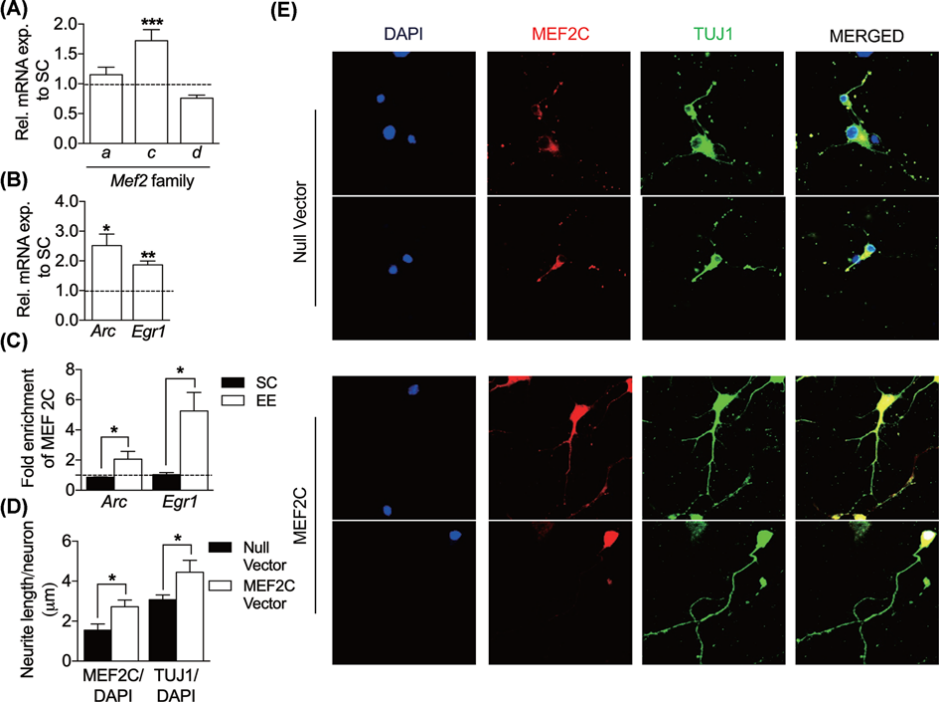
Enriched environment increases MEF2C binding at the promoter regions of immediate-early genes, *Arc* and *Egr1* and its overexpression increases TUJ1 (**A**) Relative mRNA expression of *Mef2* family transcription factors. EE up-regulates *Mef2c* mRNA expression in the visual cortex (SC, *n*=3; EE, *n*=3; two-way ANOVA Sidak’s multiple comparisons test)*.* (**B**) Mice raised in EE have a significant increase in *Arc* and *Egr1* mRNA expression compared to SC in the visual cortex (SC, *n*=5; EE, *n*=5; unpaired *t* test). Results are represented as relative mRNA to SC and normalized to two housekeeping genes, *β-actin* and *Gapdh*. (**C**) EE elevates MEF2C enrichment at *Arc* and *Egr1* promoter regions (*Arc*, SC, *n*=4; EE, *n*=3; *Egr1*, SC, *n*=3; EE, *n*=3; unpaired *t* test). ChIP-qPCR of MEF2C at the promoter regions of *Arc* and *Egr1*. Results are represented as fold enrichment of MEF2C normalized to No Antibody Control. (**D**) Quantification of neurite length in both the null vector and MEF2C-overexpressed cultures. MEF2C overexpression increases neurite length per neuron by 1.45 times (MEF2C/DAPI: Null vector, black bar, *n*=14, MEF2C, white bar, *n*=15; TUJ1/DAPI: Null vector, *n*=14, MEF2C, *n*=15, unpaired *t* test). (**E**) Representative immunocytochemistry images of MEF2C-transduced neurons and the null vector. MEF2C overexpression in neurons shows more elaborated dendritic arborization with increases TUJ1 dendritic marker. Black bars represent SC and white bars represent EE, unless specified. Data are shown as means±S.E.M., and asterisks denote statistical significance, *P<0.05, **P<0.01, ***P<0.001.

**Figure 3:**
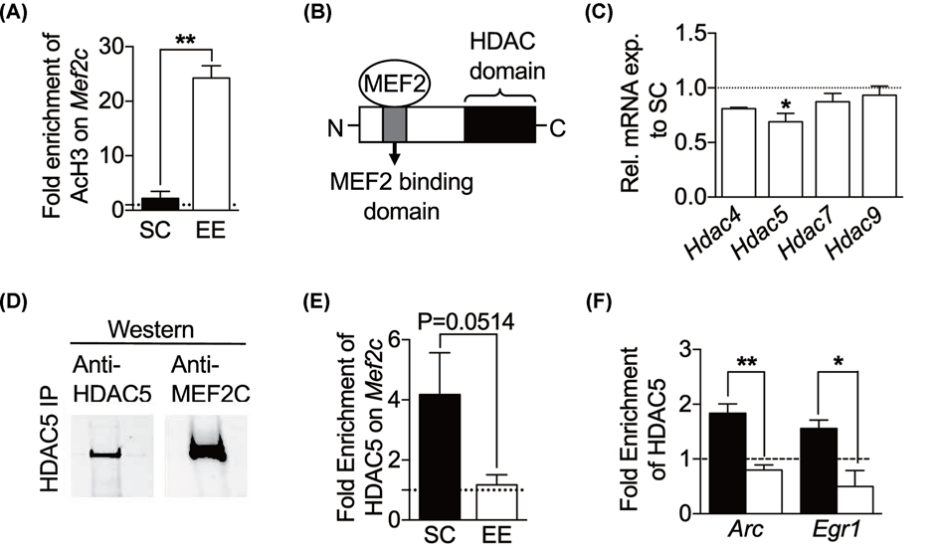
HDAC5 regulates MEF2C by a reduction in binding at *Arc* and *Egr1* promoter sites in an enriched environment (**A**) EE significantly enriches AcH3 at the *Mef2c* promoter region in the visual cortex (SC, *n*=3; EE, *n*=3, unpaired *t* test). (**B**) HDAC5 interacts with MEF2 family transcription factors. Schematic diagram of MEF2-binding site on HDAC5 showing the catalytic domain of HDAC5 located on the C terminus. (**C**) EE significantly reduces *Hdac5* expression in visual cortex when compared SC (SC, *n*=4; EE, *n*=4; two-way ANOVA Sidak's multiple comparisons test). Results are normalized to two housekeeping genes, *β-actin* and *Gapdh* and relative to SC. (**D**) MEF2C interacts with HDAC5**.** Coimmunoprecipitation of HDAC5 from cell extracts of visual cortex (*n*=3). (**E**) EE attenuates the binding of HDAC5 at the *Mef2c* promoter (SC, *n*=3; EE, *n*=3; unpaired *t* test). (**F**) EE significantly reduces HDAC5 binding at the *Arc* and *Egr1* promoter regions. ChIP-qPCR of HDAC5 on *Arc* and *Egr1* promoters (SC, *n*=3; EE, *n*=3, unpaired *t* test). Results are represented as fold enrichment of HDAC5 and AcH3 normalized to No Antibody Control. Black bars represent SC and white bars represent EE, unless specified. Data are shown as means±S.E.M., and asterisks denote statistical significance, *P<0.05, **P<0.01.

All data are represented using mean±SEM. Results between independent groups were interpreted using two-tailed unpaired *t* test unless otherwise stated. Analysis for Figures 1B,E, 2A and 3C were conducted using two-way ANOVA Sidak’s multiple comparisons test and corrected for multiple testing. Statistical analyses were performed and graphs were illustrated using GraphPad PRISM® Version 7.0a. Differences were considered statistically significant when *P*<0.05. SC and EE were represented by black and white bar respectively, unless otherwise stated.

## Results

### Environmental enrichment increases spine density and dendritic complexity of pyramidal neurons in the visual cortex

Enriched environment has been shown to increase dendritic branching, spine density and arborization in the hippocampus, striatum, occipital and motor cortex in rodents [31]. EE also promotes structural reorganizations in the brain. As dendritic spine dynamics is widely assumed to be the cellular basis for synaptic plasticity [32,33], we carried out Golgi-cox staining to visualize the dendritic morphology and dendritic spines in primary visual cortex (V1) of SC and EE mice. Indeed, it can be seen that EE dramatically increases the total and basolateral spine density with a significant increase in the apical spine density in the visual cortex of juveniles raised in EE but not in those raised in SC (Figure 1B, bottom panel) (apical spine density: SC, black bar, 0.66±0.04, *n*=3 neurons; EE, white bar, 0.95±0.06, *n*=3 neurons; *P*=0.0433, two-way ANOVA Sidak’s multiple comparisons test). Next, we assessed the morphological changes between the two environment paradigms by Golgi-impregnated neurons *in vivo*. Sholl analysis of the Golgi-stained pyramidal neurons was carried out to determine the dendritic length and branching (Figure 1D). There is no change observed in dendritic length between SC and EE. The number of intersections was evaluated in four different concentric regions: 25–50 μm, 75–100 μm, 125–150 μm and 175–200 μm from the center of the soma. Enriched animals showed a significantly higher number of intersections in the range of 75–100 μm for the apical dendrites compared to SC animals (Figure 1E) (SC, black squares, 4.50±1.04, *n*=4; EE, white squares, 8.50±1.04, *n*=4; *P*=0.0319, two-way ANOVA Sidak’s multiple comparisons test).

We also checked the amount of cytoskeletal processes, marked by TUJ1 and found that early EE increases TUJ1 expression by 2.4-fold (Figure 1C, bottom panel) (data relative to SC and normalized to β-actin; SC, black bar, 0.77±0.37, n=4; EE, white bar, 1.90±0.68, n=4, t(3)=3.69, *P*=0.0345, paired *t* test). Though it was reported that β-actin expression in cortical circuits changes during postnatal development in an activity-dependent manner [34], in our hands, β-actin did not differ from the EE and SC paradigms as seen in (Figure 1C, top panel) and the animals from the two paradigms were also age-matched. From this, we conclude that it is changes in dendritogenesis rather than neurogenesis occurring after the EE paradigm exposure.

### Environmental enrichment accelerates the organization of the visual cortex

To further substantiate our results above, eye opening of pups was monitored from PND 0 to PND 16. We observed EE pups to experience significantly earlier eye opening than SC pups, leading to earlier experiential exposure that could accelerate visual acuity (Supplementary Figure S2C) (SC, 15.50±0.29, *n*=4; EE, 14.0±0, *n*=4; t(6)=5.20, *P*=0.0020, unpaired *t* test). Although we did monitor the duration mothers spent with their pups in SC and EE, we did not measure other maternal care indicators such as licking behavior that have been found to accelerate visual acuity [19,35]. We have also conducted visual acuity assessment using visual water test to investigate if visual acuity is accelerated in EE. We noticed EE mice were faster to train compared with SC mice (Supplementary Figure S2E) (SC, 15.00±1.14, *n*=5; EE, 8.00±2.00, *n*=2; t(5)=3.21, *P*=0.0238, unpaired *t* test). Indeed, we found EE pups to achieve the same trial accuracy of 70% with significantly higher spatial frequency of 0.06 cycles/degree than SC mice (Supplementary Figure S2F) (SC, 0.56±0.01, *n*=19; EE, 0.62±0.03, *n*=8; t(25)=2.33, *P*=0.0284, unpaired *t* test). Together with the results above, we concluded that EE accelerates the organization of the visual cortex as presented in Figure 1A, where numerous neurons extend throughout the layers as opposed to SC, where neurons are mainly found in layers V and VI.

### Environmental enrichment regulates the expression of *Mef2c* transcription factor and immediate-early genes, *Arc* and *Egr1* in the visual cortex

We sought to unravel the molecular mechanism behind EE that drives these developmental changes. MEF2 family transcription factors are vastly expressed in the brain and are activated by extracellular stimuli and calcium influx in neurons [36] to trigger a cascade of gene expression responsible for synaptic plasticity. We hypothesized the changes we observed after EE may be mediated via the MEF2 transcriptional program. First, the mRNA expression levels of all the *Mef2* family members, *Mef2a, Mef2b, Mef2c* and *Mef2d* were quantified in the visual cortex. Among the *Mef2* family, only *Mef2c* expression was found to be significantly up-regulated in EE relative to SC (Figure 2A) (SC, 1.00±0.05, *n*=3; EE, 1.72±0.18, *n*=3, *P*=0.0008, two-way ANOVA Sidak’s multiple comparisons test) while the expression levels of the other members remain relatively unchanged (*Mef2a* and *Mef2d*) or below detectable levels (*Mef2b*). Sensory experience regulates the expression of immediate-early genes such as *Egr1* and *Arc* [10,37] and are increased with enhanced sensory experience [38,39]. It has been demonstrated that *Egr1* indirectly regulates synaptic plasticity via its regulation of *Arc* [40]. We investigated the transcriptional regulation of *Arc* and *Egr1* upon exposure to environmental enrichment. Mice exposed to EE show significantly increased levels of *Arc* and *Egr1* mRNA in the visual cortex (Figure 2B) (*Arc*; 2.52±0.39, *n*=5, t(6)=2.92, *P*=0.0265; *Egr1*; 1.87±0.13, *n*=5, t(6)=5.12, *P*=0.0022, unpaired *t* test).

### Environmental enrichment increases the binding of MEF2C at *Arc* and *Egr1* promoter sites in the visual cortex

Since *Arc* and *Egr1* mRNA levels were increased in EE visual cortex, we wanted to determine if their transcription were regulated by MEF2C. We performed a ChIP to pull down MEF2C, followed by qPCR and quantified MEF2C’s occupancy on the promoters of *Arc* and *Egr1*. MEF2C significantly increase in fold enrichment at the *Arc* and *Egr1* promoter regions in the EE visual cortex as opposed to SC (Figure 2C) (*Arc*: SC, black bar, 0.86±0.02, *n*=4; EE, white bar, 2.06±0.52, *n*=3, t(5)=2.75, *P*=0.040; *Egr1*: SC, 1.03±0.15, *n*=3; EE, 5.25±1.24, *n*=3, t(4)=3.40, *P*=0.0274, unpaired *t* test).

### MEF2C overexpression increases dendritic processes and arborization *in vitro*

To parallel what we had observed in the EE animals (Figure 1D), we also wanted determine if MEF2C plays a role in dendritogenesis *in vitro.* We quantified the neurite length in both the null vector and MEF2C-overexpressed cultures using ImageJ. Indeed, the neurite length per neuron increased by 1.45 times (Figure 2D) (MEF2C/DAPI: Null vector, black bar, 1.56±0.30, *n*=14, MEF2C, white bar, 2.72±0.33, *n*=15, t(27)=2.58, *P*=0.016; TUJ1/DAPI: Null vector, 3.07±0.24, *n*=14, MEF2C, 4.45±0.59, *n*=15, t(27)=2.10, *P*=0.045, unpaired *t* test) after MEF2C over expression. Thus, MEF2C-transduced neurons showed more elaborated dendritic arborization (Figure 2E, TUJ1).

### Environmental enrichment induces an open chromatin structure by increasing acetylation of histone H3 at *Mef2c* promoter region

EE induces chromatin remodeling via increased acetylation of histones H3 (AcH3) and H4 [41]. We questioned whether the increase in *Mef2c* mRNA observed earlier is due to the increased histone acetylation. To validate this, we performed a ChIP of AcH3 and quantified its enrichment at the *Mef2c* promoter. AcH3 was significantly highly enriched at the *Mef2c* promoter region in EE visual cortex (Figure 3A) (SC, black bar, 2.21±1.28, *n*=3; EE, white bar, 24.23±2.30, *n*=3, t(4)=8.38, *P*=0.0011, unpaired *t* test), indicating that EE stimulates the transcription of *Mef2c* by histone acetylation.

### Environmental enrichment down-regulates *Hdac5* and reduces its interaction with MEF2C

Next, we wanted to identify which HDAC is responsible for the increase in histone acetylation on *Mef2c*. HDAC5 has been identified as a repressor of MEF2C transcriptional activity [42–44] through the binding of HDAC5 on its MEF2-binding domain (Figure 3B). We next quantified the mRNA expression levels of *Hdac5* in both SC and EE visual cortices. *Hdac5* was significantly decreased in the EE visual cortex (Figure 3C) (EE, 0.69±0.08 relative to SC, 1.04±0.09, *n*=4 per group, *P*=0.0375, two-way ANOVA Sidak’s multiple comparisons test).

To ascertain if MEF2C and HDAC5 proteins interact with each other, we carried out a co-immunoprecipitation of HDAC5 and probed with an anti-MEF2C antibody. MEF2C indeed forms a protein-protein interaction with HDAC5 (*n*=3; Figure 3D). We also checked the occupancy of HDAC5 on the promoter of *Mef2c* gene by ChIP. Indeed, there were low levels of HDAC5 enrichment at the *Mef2c* promoter in EE visual cortex (Figure 3E) (SC, black bar, 4.18±1.39, *n*=3; EE, white bar, 1.17±0.34, *n*=3, t(4)=2.11, *P*=0.0514, unpaired *t* test).

To further prove that an inverse relationship exists between MEF2C and HDAC5, the HDAC5 immune complexes were analyzed for binding at the *Arc* and *Egr1* promoters. There was a significant reduction in HDAC5 binding at the *Arc* and *Egr1* promoters of EE visual cortex (Figure 3F) (*Arc*: SC, black bar, 1.84±0.17; EE, white bar, 0.80±0.09, *n*=3 per group, t(4)=5.29, *P*=0.0061; *Egr1*: SC, black bar, 1.56±0.15; EE, white bar, 0.50±0.29, *n*=3 per group, t(4)=3.23, *P*=0.0321, unpaired *t* test). The qPCR analysis for the HDAC5 ChIP was inversely correlated to that of the MEF2C ChIP, suggesting a relationship between these two proteins.

**Figure 4:**
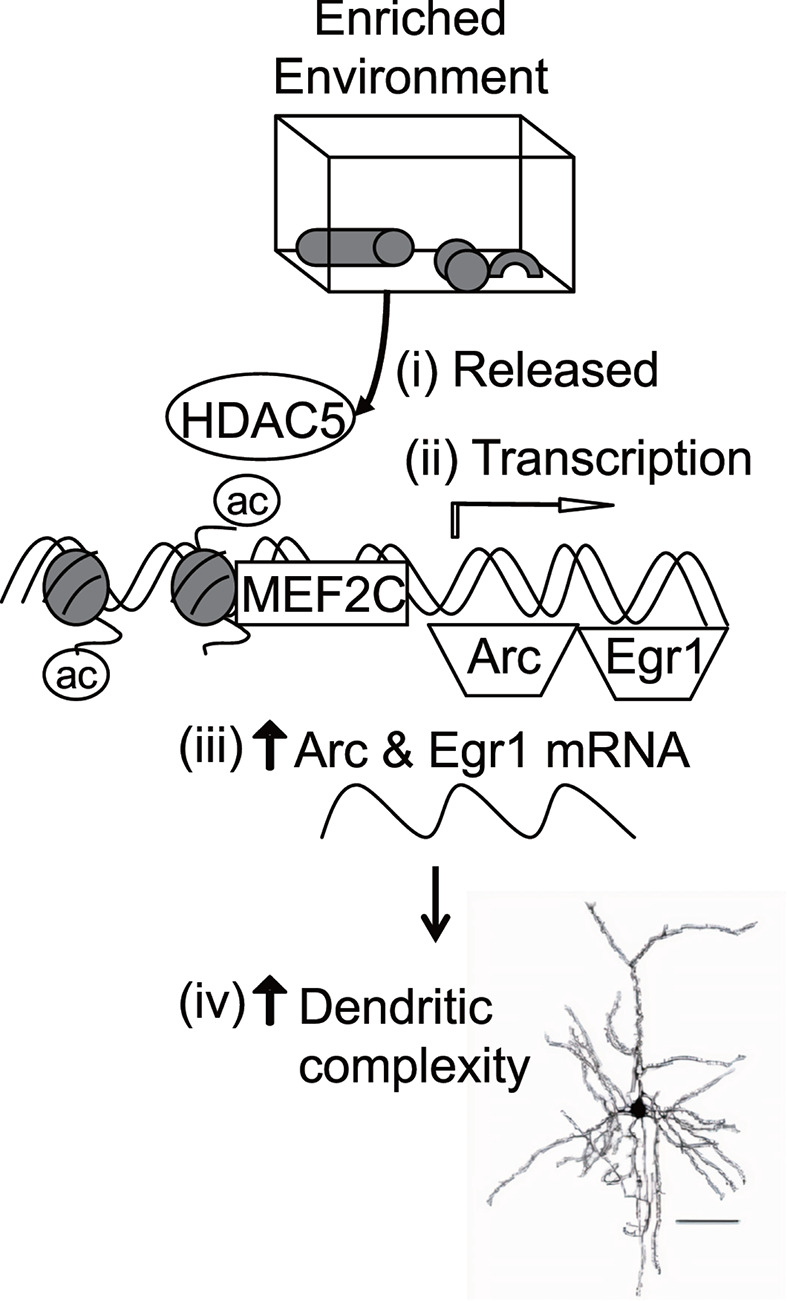
MEF2C repression by HDAC5 is overcome in mice raised in an enriched environment leading to the transcription of *Arc* and *Egr1* genes Schematic representation of the proposed molecular mechanism underlying enriched environment. In standard condition, HDAC5 binds to MEF2C and represses its function as a transcription factor. EE stimulates visual cues to activate a cascade of events leading to an increase in dendritic complexity. (**i**) Visual stimuli releases HDAC5 thereby activating MEF2C transcription factor. This activates the transcription of *Mef2c.* (**ii**) MEF2C binds to promoters of immediate-early genes, *Arc* and *Egr1* and activates their transcription. (**iii**) Increase in mRNA expression of *Arc* and *Egr1*. (**iv**) *Arc* and *Egr1* activation via MEF2C may lead to the increase in dendritic spine density seen in the early EE mice.

Together, our findings demonstrated the close interaction of MEF2C and HDAC5 in different environmental paradigms and illustrated in Figure 4. In standard condition, HDAC5 represses MEF2C function by forming an HDAC5-MEF2C protein complex in mouse visual cortex. This complex prevents MEF2C from activating the transcription of the immediate-early genes. By placing mice in EE, this repression is removed as (i) HDAC5 is released from MEF2C. This (ii) increases the acetylation of histone H3 levels at the *Mef2c* promoter, leading to its transcription. Activated MEF2C functions as a transcription factor and (iii) initiates the transcription of *Arc* and *Egr1* genes corresponding to their increase in mRNA levels in EE visual cortex as seen earlier. *Arc* and *Egr1* may contribute to (iv) the increase in total and apical dendritic spine density observed in early EE mice (Figure 1B).

## Discussion

Despite numerous studies involving EE, the molecular mechanisms underlying its efficacy are still poorly understood. Here, we demonstrated that (1) EE increases dendritic spines tremendously during the CP; EE induces (2) an increase in *Arc* and *Egr1* expression levels and their expression is regulated by MEF2C; MEF2C transcriptional activity can be repressed via its interaction with HDAC5 and (4) in EE, the reduction in HDAC5 decreases its occupancy on promoters of *Arc* and *Egr1*. This is the first demonstration of the epigenetic dynamics of MEF2C and HDAC5 targeting on *Arc* and *Egr1* genes to regulate experience-dependent plasticity.

External stimuli sculpts overall neural circuitry and hence its plasticity. In the clinical setting, EE is being used as a non-invasive treatment for amblyopia [35,45]. Visual enrichment, such as playing of video games, has been used to treat amblyopic adults [46,47]. Incorporation of exercise and visuomotor engagement into existing therapy have drastically improved conditions in amblyopic patients [48]. Physical stimulation with body massage has been found to improve visual function in pre-term infants [46]. Given the growing body of evidence of the beneficial treatment of EE in visual deficits, we sought to unravel the underlying physiological and molecular mechanisms of EE and how it improves neural circuitry.

To unravel the molecular basis of EE that led to the increased visual acuity observed, we first carried out a motif-scanning approach on online available EE- related GEO database to predict gene regulatory sequences across the genome. MEF2 proteins have been shown to be key regulators of synapse development and function and their activity are dependent on neuronal stimuli, such as neurotrophins or calcium influx [36]. We hypothesized that the MEF family may be activated in EE to drive the observed developmental changes in Figure 2A and regulate activity-dependent transcription after EE. Indeed, our *in silico* analysis agreed with the genome wide analysis study [36,49]. In the present study, we found significant increase of *mef2c* expression in EE mice. Notably, MEF2C is one of the most highly expressed isoforms [50], crucial for learning and memory formation [51]. MEF2C overexpression in adult prefrontal cortex has been found to improve cognition [52]. *Mef2c* is also a candidate risk gene for various neurodevelopmental disorders such as schizophrenia [52], major depressive disorder [53] and Alzheimer’s disease [54]. MEF2C haploinsufficiency and embryonic studies have reported reduce neurogenesis, increase neuronal cell death and affect excitatory to inhibitory balance [55,56].

We next used a candidate gene approach to identify gene targets of MEF2 family transcription factors, narrowing the selection to *Arc* and *Egr1*. Previous studies demonstrated EE rats have elevated *Arc* and *Egr1* mRNA [38,39,57,58], supporting our gene selection. Research associating *Arc* with visual acuity has emerged in recent years. *Arc* gene produces a protein important for memory consolidation, memory formation, and visual cortical development [59,60]. Functionally, *Arc*-/- mice exhibited deficits in visual cortical plasticity [61] and visual restoration triggered by *Arc* transcription was shown in both adult congenital blind [62] and wild-type mice [63]. These results establish the importance of normal ocular dominance during CPs [64]. EGR1 is a transcription factor and its expression has been found to be regulated by synaptic stimulation and visual plasticity in the visual cortex [65,66]*.* Monocular visual stimulation has been found to induce and restore *Egr1* expression and protein levels during CPs [67–69]. However, loss of *Egr1* does not necessarily dictate experience-dependent plasticity [66]. The relationship between MEF2C and both *Erg1* and *Arc* is evident, whereby MEF2 shRNA knockdowns attenuate *Arc* expression levels [70], activation of MEF2 promotes *Arc* [49] and MEF2 was found to facilitate *Arc* expression in activity dependent neuronal plasticity in the visual cortex [71]. In order to demonstrate their interaction, we used ChIP assay and there was indeed an association between MEF2C on *Arc* and *Egr1* genes in EE conditions. This could be explained by stimulation-transcription coupling, where EE potentially stimulates neurons to induce *Arc* and *Egr1* gene expressions via MEF2C and regulate synaptic plasticity during CPs [72]. We then show the importance of MEF2C in dendritogenesis. MEF2 proteins have been found to trigger the dendritic development and survival. Significant dendritogenesis was shown after TAM administration in *Mef2c* KO mice [73]. MEF2C overexpression in cultures showed increases neurite length and protein expression of dendritic marker TUJ1. This suggests that MEF2C plays a crucial role in early dendritic development and differentiation during CPs.

The activity of MEF2 gene is regulated by the dissociation of class IIa HDACs from its DNA-binding domain [43,44,74,75] and we depicted their interaction in Figure 4. The activation of MEF2C could be mediated through HDAC5 phosphorylation either by calcium/calmodulin-dependent protein kinase or mitogen-activated protein kinase signaling to export HDAC5 out of the nucleus and prevent its association with DNA-binding domain to initiate neuronal differentiation [74,76]. HDAC5 down-regulation has been found to initiate neurite growth by MEF2C/M6a signaling pathway [44]. To validate the interaction, we investigated and found that EE attenuates HDAC5 binding at the *Mef2c* promoter and also the *Arc* and *Egr1* promoter regions. Given the HDAC5–MEF2C interaction and its association to regulate neurite development, we would like to address the signaling cascades of EE via HDAC5 and MEF2C and understand the role of MEF2C and HDAC5 in visual plasticity.

MEF2C protein expression are found to be crucial for visual neuroplasticity during critical period [77] and this has been shown in our study. Enriched environment is found to restore visual acuity in monocular deprived animals during CPs, and given the prominent interaction of MEF2C on *Arc* and *Egr1* genes during critical period, there is a possible association between MEF2C activation and visual acuity [13,78]. To further strengthen our view, we would like to overexpress *Mef2c* in deprived knockdown adult mice to see an improvement in their visual acuity.

## Supplementary Material

Supplementary Figures S1-S2

## Abbreviations

ChIP: chromatin immunoprecipitation
*Egr1*: early growth response protein 1
*Mef2c*: myocyte enhancer-binding factor 2C
*Arc*: activity-regulated cytoskeleton-associated protein
HDAC: histone deacetylase
EE: enriched environment
SC: standard condition
