# Supplementary material for: MEF2C and HDAC5 regulate Egr1 and Arc genes to increase dendritic spine density and complexity in early enriched environment
Source: Neuronal Signal. 2020 Jul 23;4(3):NS20190147. doi: 10.1042/NS20190147 (PMC7378308; doi:10.1042/NS20190147)
Supplement: Supplementary Figures S1-S2 [file NS-2019-0147C_supp.pdf]

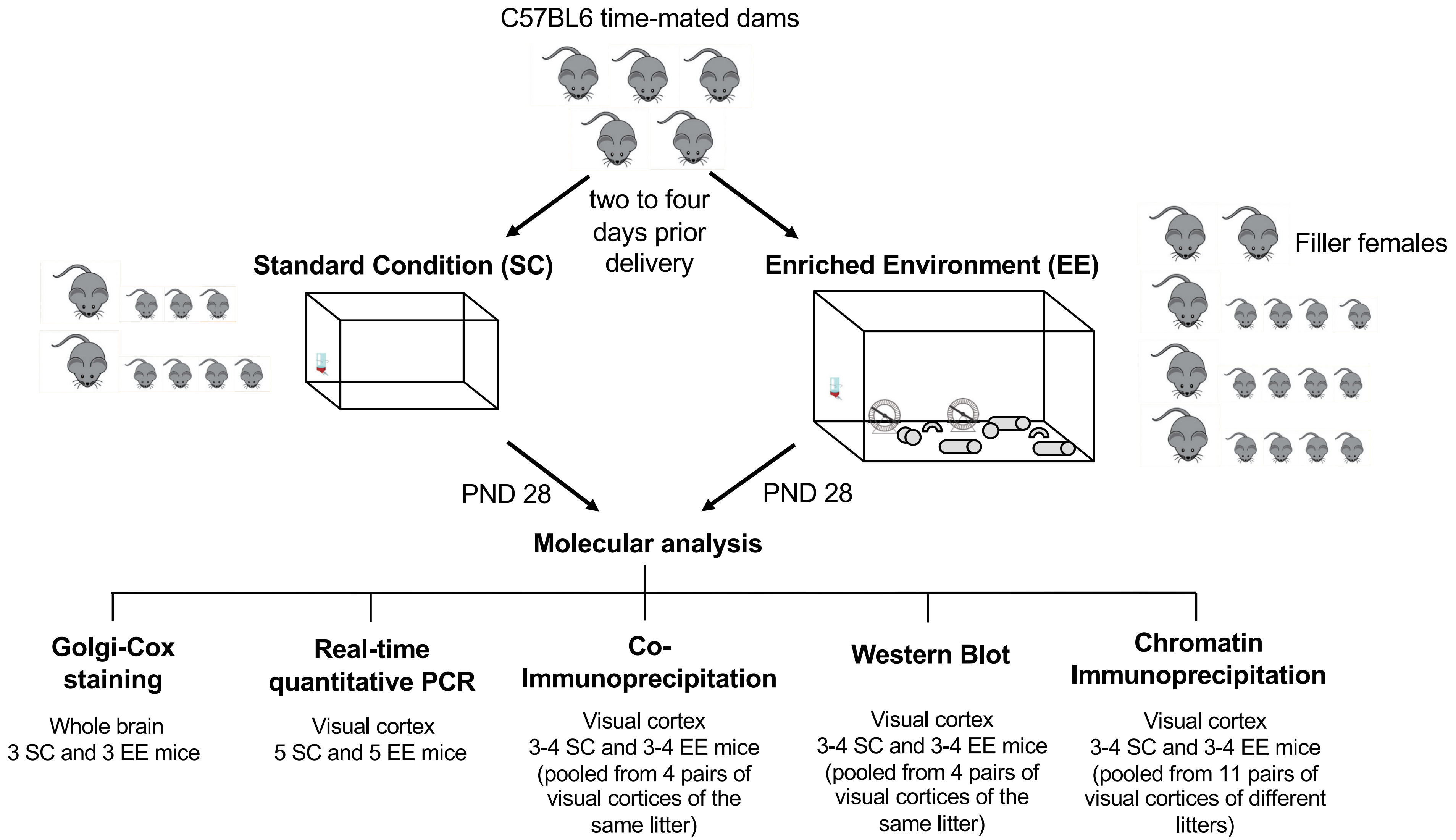

Puang et al Supplementary Figure S1

Supplementary Legends

Figure S1. A schematic representation of rearing environments and molecular analysis conducted. C57BL6 time-mated dams were placed in SC or EE for two to four days prior to the estimated time of delivery and subsequently gave birth in their respective environments – SC and EE. A standard condition was a basic housing of a 19 x 30cm shoebox without any form of social stimulation. The enriched environment consists of a 45 x 45 cm cage arena with enhanced living conditions, containing various toys such as running wheels, tunnels, toilet rolls, wood chews, shelters and nesting material. The positions of the toys were changed every week and two filler females are part of EE to promote social interaction. The pups were raised in the respective environments with their mothers from birth to the peak of the critical period or postnatal day 28 (PND 0-28). The pups were sacrificed on PND 28 for molecular analyses. A summary of the molecular analysis, together with the brain region and mice used, was listed.

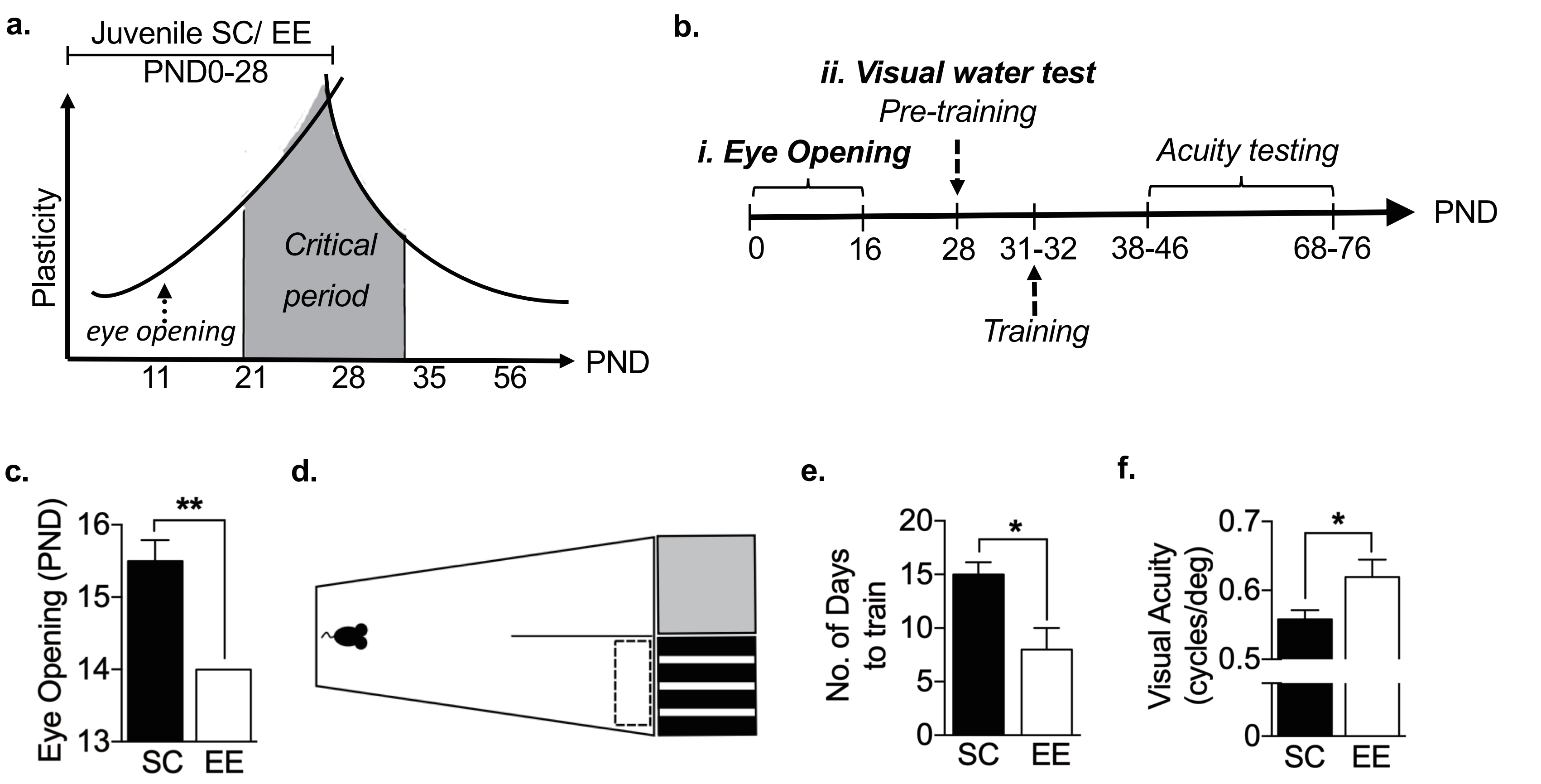

Puang et al Supplementary Figure S2

Figure S2. Environmental enrichment at critical period shown to accelerate visual acuity.

a. Critical period of the mouse visual cortex. Animals were exposed to SC and EE conditions respectively, from the start to the peak of the critical period, PND0-28. **b.** Experimental timeline for eye opening and visual water task from pre-training, beginning from P28, to testing. i. Eye opening was conducted for both SC and EE pups from PND0-16. ii. Visual water test was conducted for selected SC and EE pups. Pre-training usually takes 3-4 days, training takes up to two weeks and testing takes up to a month. **c.** Eye opening observations in SC and EE mice. Eye opening is defined as a tear in the ocular membrane, resulting in obvious presentation of the eye. Mice placed in EE (white bar) were observed a significantly earlier eye opening compared to SC (black bar). (SC, n=4; EE, n=4; unpaired *t* test). **d.** A schematic representation of the visual water task for visual acuity from Acumen. Two monitor screens presented either vertical gratings or the equiluminant grey stimulus at one end of the tank. A hidden platform (dashed rectangle) was present in the channel with gratings. **e.** EE mice completed the visual water task training in a shorter period of time compared to SC mice. (SC, n=5; EE, n=2; unpaired *t* test). **f.** Effect of EE and SC mice on visual performance. Animals were trained on the visual water task to measure their visual acuity. EE mice have a significantly higher visual acuity than SC (SC, n=19; EE, n=8; unpaired *t* test). Acuity is represented as a mean of visual performance in each group. Data are shown as means$\pm$s.e.m., and asterisks denote statistical significance by unpaired *t* test.
